# Molecular identification of four *Sarcocystis* species in cattle from Lithuania, including *S*. *hominis*, and development of a rapid molecular detection method

**DOI:** 10.1186/s13071-020-04473-9

**Published:** 2020-12-07

**Authors:** Petras Prakas, Živilė Strazdaitė-Žielienė, Vytautas Januškevičius, Francesco Chiesa, Agnė Baranauskaitė, Eglė Rudaitytė-Lukošienė, Elena Servienė, Saulius Petkevičius, Dalius Butkauskas

**Affiliations:** 1 Nature Research Centre, Vilnius, Lithuania; 2 Lithuanian University of Health Science, Kaunas, Lithuania; 3 Department of Veterinary Science, University of Turin, Turin, Italy

**Keywords:** Cattle, *Sarcocystis hominis*, Trypsin digestion, Molecular identification, *cox*1, 18S rRNA gene

## Abstract

**Background:**

Six *Sarcocystis* species are known to use cattle (*Bos taurus*) as the intermediate host, two of which, *S*. *hominis* and *S*. *heydorni*, are zoonotic. There is a need for a method that will enable rapid identification of the *Sarcocystis* species in cattle.

**Methods:**

The diaphragm muscles of 102 cattle from Lithuania were examined for the presence of *Sarcocystis* spp., using two different methods for species identification. Individual sarcocysts were isolated from squash preparations of the diaphragm muscle under the light microscope, followed by genetic characterisation of excised cysts using sequence analysis of the 18S rRNA (*18S* rRNA) and cytochrome c oxidase subunit I (*cox*1) genes. The same cattle muscle samples were digested and species-specific PCR analyses targeting *cox*1 were developed to identify the *Sarcocystis* isolates to the species level.

**Results:**

Under the light microscope, sarcocysts were detected in 87.3% of animals, and *Sarcocystis* infection was verified in all digested samples. Three species, namely *S*. *cruzi* (*n* = 20), *S*. *bovifelis* (*n* = 23) and *S*. *hirsuta* (*n* = 6), were identified by DNA sequence analysis of isolated sarcocysts. Based on sequence analysis of *cox*1, the level of genetic variability depended on *Sarcocystis* species and geographical location. Four *Sarcocystis* species, *S*. *cruzi* (96.1%), *S*. *bovifelis* (71.6%), *S*. *hirsuta* (30.4%) and *S*. *hominis* (13.7%), were confirmed in the digested samples. In individual samples, the most common finding was two species of *Sarcocystis* (44.1%), followed by three species (26.5%), a single species (24.5%) and four species (4.9%).

**Conclusions:**

Although examination of tissue preparations under the light microscrope did not detect any sarcocysts belonging to *S*. *hominis*, this species was identified in the digested samples subjected to a *cox*1-specific PCR analysis. These results demonstrate the need for effective molecular diagnosis techniques to detect *Sarcocystis* spp., which may be present at a lower prevalence and not detectable among the limited number of sarcocysts identified individually under the light microscope. 
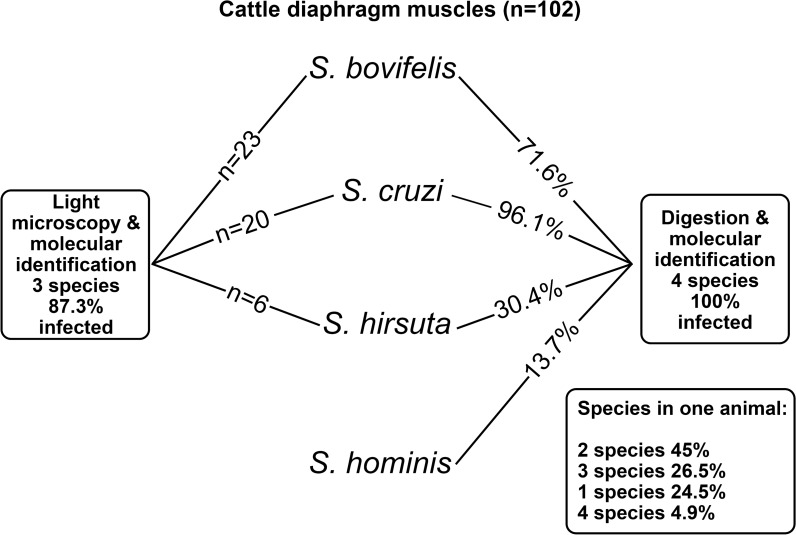

## Background

Protozoan parasites of the genus *Sarcocystis* (Apicomplexa: Sarcocystidae) infect mammals, birds and reptiles. The genus is characterised by an obligatory two-host life-cycle and endogenous sporulation. These parasites form sarcocysts mainly in the muscle tissues of the intermediate hosts and develop oocysts and sporocysts in the small intestine of the definitive hosts [1].

Three *Sarcocystis* species, *S*. *suihominis*, *S*. *hominis* and *S*. *heydorni*, are known to infect humans as definitive hosts [2–4]. The latter two species can be found in the muscles of cattle (*Bos taurus*), while humans may become infected by consuming raw/undercooked beef containing mature sarcocysts of *S. hominis* and *S. heydorni*. There has been much debate about the validity and classification of several *Sarcocystis* species from cattle, as well as their specificity to the intermediate host [5–8]. Current consensus is that that cattle may harbour up to six *Sarcocystis* species, namely *S*. *bovifelis*, *S*. *bovini*, *S. cruzi*, *S*. *heydorni*, *S*. *hirsuta* and *S*. *hominis* [4, 8–11]. As morphological analysis is not commonly sufficient to identify *Sarcocystis* species in cattle [12–14], the need for molecular methods is increasing. *Sarcocystis* spp. from cattle have been molecularly characterised at the genes for 18S rRNA (*18S* rRNA), 28S rRNA (*28S* rRNA) and cytochrome c oxidase subunit I (*cox*1) and at nuclear rDNA internal transcribed spacer 1 (ITS1) [8, 10, 11, 15]. Databases contain many *18S* rRNA gene sequences of the genus *Sarcocystis* used for species identification [16–18]. However, studies have revealed that *cox*1 is the preferable target to differentiate taxonomically related *Sarcocystis* spp. whose intermediate hosts are such ruminants as cattle, sheep, goats, deer and others [19].

In Lithuania, the prevalence of *Sarcocystis* infection ranges from 44.9 to 98.1%, and infection has been recorded in six different muscle types of cattle [20]. However, the parasite species in the area under investigation have not yet been identified. In the study reported here, we isolated individual sarcocysts from squash preparations of muscle and then characterised the sarcocysts based on sequence analysis of *18S* rRNA and *cox*1. We also report out development of a diagnostic technique to identify *Sarcocystis* species in cattle based on tissue digestion and PCR analysis using species-specific primers targeting *cox*1.

## Material and methods

### Samples and microscopic examination of sarcocysts

In 2017–2018, the diaphragm muscles of 102 cattle were collected from farmers and meat processing plants from all over Lithuania. The prevalence and density of *Sarcocystis* infection were evaluated in methylene blue-stained muscle specimens by counting sarcocysts per gram of sample according to Kirillova et al. [21]. The morphological analysis of sarcocysts was performed in freshly squashed preparations as described by Prakas et al. [22]. Sarcocysts were isolated from muscle fibres under a Nikon ECLIPSE 80i light microscope (Nikon Corp., Tokyo, Japan), at 40× or 100× magnification, and morphologically differentiated according to size and shape of sarcocysts, as well as the structure of the sarcocyst wall. Overall, 49 sarcocysts excised from the muscle tissues were separately preserved in 70% ethanol for molecular analysis.

### Molecular analysis of sarcocysts

Genomic DNA was extracted from individual sarcocysts using the QIAamp® DNA Micro Kit (Qiagen, Hilden, Germany) tissue protocol according to the manufacturer’s recommendations. *Sarcocystis* species were identified by sequence analysis of *18S* rRNA and *cox*1. The SarCF/SarDR primer pair was applied for* 18S* rDNA sequence amplification [23], while the SF1/SR9 primer pair was used to amplify *cox*1 sequences [19, 24]. The PCR conditions and product evaluation were as described previously [25]. The samples were sequenced directly with the 3500 Genetic Analyzer (Applied Biosystems, Thermo Fisher Scientific, Foster City, CA, USA) using the same forward and reverse primers as for the PCR. The obtained sequences were compared with those of various *Sarcocystis* spp. by Nucleotide BLAST (megablast option). A total of 213 *cox*1 and 126* 18S* rRNA sequences were selected and aligned using the MUSCLE algorithm [26] included in the MEGA7 software [27]. TOPALi v2.5 software [28] was used to select a nucleotide substitution model with the best fit to the aligned sequence datasets and to construct phylogenetic trees under Bayesian inference. The intraspecific genetic variation indices, including the number of haplotypes (h), the number of segregating sites (S), haplotype diversity (Hd) and nucleotide diversity (π), were calculated for whole dataset and separate samples using DnaSP v6 [29]. Genetic differentiation for* Sarcocystis* spp. sample pairs was assessed with* Φ*_ST_ (i.e. the proportion of genetic diversity due to differences among populations) based on the Tamura-Nei distance [30] with Arlequin v. 3.5.2.2 [31]. The statistical significance of* Φ*_ST_ was tested by 10,000 permutations and at the 0.05 confidence level.

### Trypsin digestion

Digestion of cattle diaphragm muscles was performed according to the optimised protocols of Verma et al. [32] and Chiesa et al. [33]. Following trypsin digestion of 20-g samples each in 50 ml of digestion solution (trypsin 1:250, 2.5 g/l phosphate buffered saline [PBS]) for 16 h at 37 °C in an incubator with stirring, the suspension was centrifuged for 5 min at 7000 rpm, following which the pellet was resuspended in 5 ml PBS and centrifuged for 3 min at 5000 rpm. The resulting pellet was resuspended in 5 ml PBS, and 300 µl of the suspension was used for DNA extraction.

### Molecular analysis of digested samples

Genomic DNA was extracted from the digested samples using the GeneJet Genomic DNA Purification Kit (Thermo Fisher Scientific Baltics, Vilnius, Lithuania) according to the manufacturer’s protocol. The resulting DNA samples were kept frozen at − 20 °C until used as templates for PCR amplification of *cox*1.

Primers targeting *cox*1 were designed with the help of Primer3Plus [34]. The primers designed for *S. cruzi*,* S. bovifelis*,* S. bovini*, *S. heydorni*,* S. hirsuta* and *S. hominis* are listed in Table 1. Each PCR was performed in a final volume of 25 μl containing 0.5 μM of each primer, dNTP mix (0.2 mM of each), 10× DreamTaq buffer with 20 mM MgCl_2_, 1.25 U DreamTaq polymerase (Thermo Fisher Scientific Baltics), 1 μg genomic DNA and nuclease-free water. The cycling conditions began with one cycle at 95 °C for 5 min, followed by 40 cycles at 94 °C for 45 s, 56 °C for 45 s and 72 °C for 45 s, and ending with one cycle at 72 °C for 10 min. PCR products were visualised by 1% agarose gel electrophoresis. The selected PCR products were purified using the GeneJET PCR Purification Kit (Thermo Fisher Scientific Baltics) according to the manufacturer’s recommendations. Sequencing of the purified PCR fragments was performed, and the obtained sequences were compared by BLAST analysis. Differences in the prevalence of the identified *Sarcocystis* species were evaluated using Chi-squared test.

**Table 1:** Oligonucleotide primers used to amplify *cox*1 of *Sarcocystis* spp. isolated from cattle

Primer name	Orientation	Primer sequence (5′→3′)	Species	Amplified product (bp)
GaBfEF	Forward	ATCAACTTCCTAGGTACAGCGGTATT	*S*. *bovifelis*	523
GaBfER	Reverse	CCACATCATTGGTGCTTAGTCTAGTA		
GaBnEF	Forward	ATGGAGATGTGGTATTCTGTACGTCT	*S*. *bovini*	485
GaBnER	Reverse	ACAGTGCAACATCATTGGTATGTATC		
GaCrEF	Forward	GCTATGTATCTACTTACGGCAGGTATC	*S*. *cruzi*	531
GaCrER	Reverse	GAATATAATGGCCCAGGTAAATAATG		
GsSheyF	Forward	GGTATCCGGTATGAAGCATACAAC	*S*. *heydorni*	443
GsSheyR	Reverse	GATCCGCTGTCAGTGTACGATATT		
GaHiEF	Forward	GTTGTGCGGTATGAATTATCAACCT	*S*. *hirsuta*	513
GaHiER	Reverse	GGTAAGAACTGGAATGGTTAATATCAG		
GaHoEF	Forward	TCTCTGGTTTTGGTAACTACTTCGT	*S*. *hominis*	551
GaHoER	Reverse	CAGACACTGGGATATAATACCGAAC		

## Results

### Morphological examination of sarcocysts

Examination of methylene blue-stained squash preparations of diaphragm muscles from cattle reared in Lithuania revealed the presence of sarcocysts in 87.3% (89/102) of the samples collected. The number of sarcocysts per gram of muscle ranged from one to 79 (mean 10.5/g muscle, median 5.0/g muscle). Three types of sarcocysts were detected under the light microscope. Type I sarcocysts were thread-shaped, about 800 × 75 μm in size (*n* = 58) and had thin 3-µm-long hair-like protrusions; these sarcocysts were characterised as being *S*. *cruzi*-like. Type II sarcocysts were spindle- or fusiform-shaped, about 1600 × 130 μm in size (*n* = 40) and had 5- to 6-µm-long finger-like protrusions; these cysts were characterised as being *S*. *bovifelis*- and *S*. *bovini*-like. Type III sarcocysts were thread-shaped, about 2600 × 500 µm in size (*n* = 2) and had about 7-µm-long finger-like protrusions; these cysts were considered as being *S*. *hirsuta*-like.

The molecular analysis revealed that cysts belonging to types I–III were sarcocysts of *S*. *cruzi* (*n* = 20), *S*. *bovifelis* (*n* = 23) and *S*. *hirsuta* (*n* = 6), respectively. In some cases, sarcocysts morphologically assigned to* S. hirsuta* were identified as *S*. *bovifelis* or *vice versa*. In two cases, fragments of sarcocysts appeared to be smooth; however, based on molecular methods these cysts were assigned to *S*. *cruzi*.

### Molecular results on isolated sarcocysts

The obtained* 18S* rRNA sequences of* S. bovifelis* (865 bp), *S*. *cruzi* (867 bp) and *S*. *hirsuta* (874 bp) were deposited in GenBank under accession numbers MT792432–MT792480. The analysed* 18S* rRNA fragments correspond to position 924–1774 of the *S*. *neurona* (U07812) sequence. Comparison of the* 18S* rRNA sequences of *S*. *bovifelis* and *S*. *cruzi* did not reveal intraspecific genetic variability. In contrast, one transition (C/T) at nucleotide position 588 was detected between the obtained isolates of *S*. *hirsuta*. Twenty sequences of *S*. *cruzi* showed 99.8–100% identity with other sequences of this species available in GenBank (AB682779–AB682780, AF017120, JX679467–JX679468, KC209738, KT901167, LC171827–LC171830). Among the *Sarcocystis* spp. isolated from cattle, the* 18S* rRNA sequences of *S*. *cruzi* demonstrated the greatest similarity (99.1–99.2%) with *S*. *heydorni* (KX057996–KX057997). The BLAST analysis revealed that six* 18S* rRNA gene sequences of *S*. *hirsuta* obtained in the present study shared 99.3–100% identity with those of *S*. *hirsuta* (AF017122, JX855283, KC209741, KT901156–KT901166, LC171839, MT706003–MT706004) and showed < 98% similarity with those of other *Sarcocystis* spp. from cattle. Based on* 18S* rRNA sequence analysis, Lithuanian isolates of *S*. *bovifelis* displayed 99.8–100% identity with *S*. *bovifelis* (KT901117–KT901138, KC209742–KC209744) and 99.2–99.5% similarity with *S*. *bovini* (KT901139–KT901155). It should be noted that these two species could be separated based on three fixed nucleotide positions: at nucleotide positions 441, 457 and 587 of the region studied, *S*. *bovifelis* had A, T and A, and *S*. *bovini*—G, A and T, respectively.

**Fig. 1:**
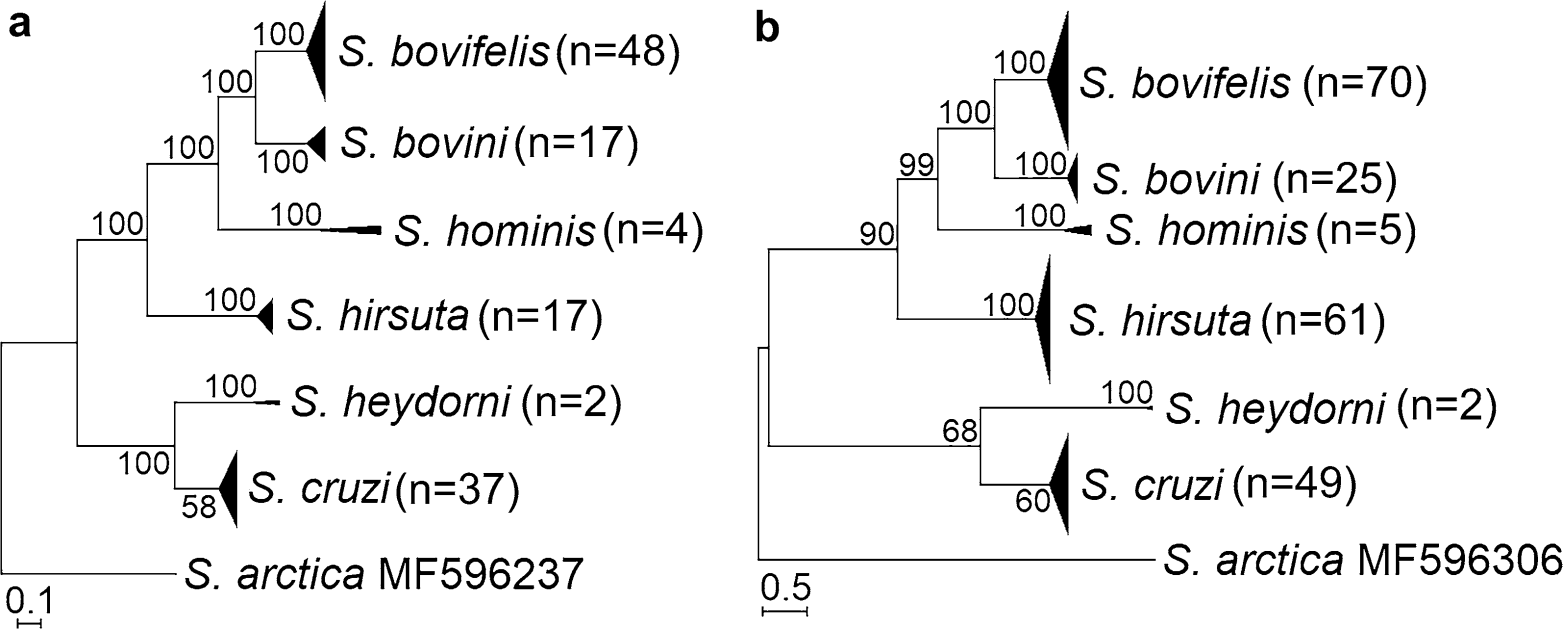
The phylogenetic placement of *Sarcocystis* spp. from cattle based on sequence analysis of the 18S rRNA (*18S* rRNA; **a**) and cytochrome c oxidase subunit I (*cox*1; **b**) genes. Trees were scaled according to the branch length and rooted on *S*. *arctica*

Phylogenetic analysis showed that the* 18S* rRNA fragment used in this study was suitable for the discrimination of *Sarcocystis* spp. forming cysts in the muscles of cattle (Fig. 1a). It should be noted that four species (*S*. *bovifelis*, *S*. *bovini*, *S*. *hominis* and *S*. *hirsuta*) characterised on the basis of sarcocysts with finger-like protrusions [8] were grouped into one cluster. The same topology was shown by the phylogenetic tree generated by sequence analysis of *cox*1 (Fig. 1b).

The genetic comparison of *cox*1 sequences of *S*. *bovifelis*, *S*. *cruzi* and *S*. *hirsuta* obtained in the present study are presented in Table 2. The highest sequence differences were found within *S*. *cruzi*. The *18S* rRNA region used in this study was characterised by minimal intraspecific genetic variability among the analysed *Sarcocystis* species. Therefore, *S*. *bovifelis*, *S*. *cruzi* and *S*. *hirsuta* from different geographical areas were compared only for *cox*1. The highest overall intraspecific genetic variability was detected in *S*. *cruzi*, while a similar level of overall genetic variation was calculated in *S*. *bovifelis* and *S*. *hirsuta* (Table 3). Differences in the level of genetic variation were observed between samples of the same species. Relatively low variation was established for *S*. *bovifelis* from Lithuania and *S*. *hirsuta* from New Zealand, and relatively high variation was noticed for *S*. *cruzi* from China. The overall Φ_ST_ values showing genetic differentiation between populations were high for *S*. *hirsuta* (*Φ*_ST_ = 0.401, *P *< 0.001) and *S*. *cruzi* (*Φ*_ST_ = 0.255,* P* < 0.001) and medium for *S*. *bovifelis* (*Φ*_ST_ = 0.141, *P *< 0.01).

**Table 2:** Molecular examination of *Sarcocystis* species from cattle based on *cox*1 sequence analysis

*Sarcocystis* species	Sample type	*Cox*1 GenBank accession no. (length in bp)	Differences between obtained sequences (%)	Sequence identity (%)	
				Comparison of isolates of the same species	Comparison of isolates with other closely related species
*S*. *bovifelis*	Sarcocyst	MT796903–MT796925 (1038)	0–1.0	98.4–100% to *S*. *bovifelis* (KT900961–KT900998, KC209690–KC209696, MK962347–MK962348)	93.0–93.9% to *S*. *bovini* (KT900999–KT901022, LC171858)
	Digested	MT796952–MT796954 (471)	0	97.9–100% to *S*. *bovifelis*	92.4–93.0% to *S*. *bovini*
*S*. *cruzi*	Sarcocyst	MT796926–MT796945 (1038)	0.2–1.2	96.1–99.9% to *S*. *cruzi* (KC209597–KC209600, KT901078–KT901095, LC171859–LC171862, MG787071–MG787076)	92.6–93.9 to *S*. *levinei* (KU247874–KU247885, MH255771–MH255781)
	Digested	MT796955–MT796957 (478)	0–0.4	97.7–99.8% to *S*. *cruzi*	< 95% similarity with other *Sarcocystis* spp.
*S*. *hirsuta*	Sarcocyst	MT796946–MT796951 (1038)	0–0.7	99.2–100% to *S*. *hirsuta* (KC209634, KT901023–KT901077, LC171863)	93.4–93.7 to *S*. *buffalonis* (KU247868–KU247873, MG792800–MG792802)
	Digested	MT796958–MT796960 (461)	0–0.2	99.4–100% to *S*. *hirsuta*	95.6–96.3% to *S*. *buffalonis*
*S*. *hominis*	Digested	MT796961–MT796964 (501)	0	98.2–98.6% to *S*. *hominis* (MH021119, MK497840–MK497843)	< 90% similarity with other *Sarcocystis* spp.

**Table 3:** Measurements of intraspecific genetic variability of three *Sarcocystis* species from cattle based on *cox*1 sequence analysis

*Sarcocystis* species	Country of origin	GenBank	*n*/h	S	Hd ± SD	π ± SD
*S*. *bovifelis*	Argentina	KC209690–KC209696, KT900961–KT900993	40/17	25	0.937 ± 0.018	0.00525 ± 0.00029
	Germany	MK962348, KT900994–KT900998	6/5	17	0.933 ± 0.122	0.00635 ± 0.00281
	Lithuania	MT796903–MT796925	23/12	19	0.779 ± 0.091	0.00275 ± 0.00064
	Overall		69/32	45	0.919 ± 0.025	0.00491 ± 0.00043
*S*. *cruzi*	Argentina	KC209597–KC209600, KT901078–KT901095	22/13	23	0.931 ± 0.036	0.00687 ± 0.00093
	China	MG787071–MG787076	6/6	50	1.000 ± 0.096	0.02667 ± 0.00390
	Lithuania	MT796926–MT796945	20/20	33	1.000 ± 0.016	0.00718 ± 0.00054
	Overall		48/38	76	0.984 ± 0.010	0.01124 ± 0.00170
*S*. *hirsuta*	Brazil	KT901042–KT901056	15/5	5	0.810 ± 0.059	0.00256 ± 0.00028
	Germany	KT901057–KT901077	21/6	10	0.795 ± 0.051	0.00399 ± 0.00037
	Lithuania	MT796946–MT796951	6/5	9	0.933 ± 0.122	0.00308 ± 0.00084
	New Zeeland	KT901023–KT901041	19/2	5	0.199 ± 0.112	0.00096 ± 0.00054
	Overall		61/15	19	0.863 ± 0.028	0.00387 ± 0.00029

*n*/h, Number of isolates/number of haplotypes; S, number of segregating sites; Hd, haplotype diversity;* π*, nucleotide diversity; SD standard deviation

### Molecular results of digested samples

*Cox*1 fragments amplified using the GaBfEF/GaBfER, GaCrEF/GaCrER, GaHiEF/GaHiER and GaHoEF/GaHoER primer pairs were examined by DNA electrophoresis. To ascertain whether the used primers were species-specific, three PCR products amplified with each primer pair were purified and sequenced. Primer-annealing sequences were not included in the BLAST analysis. The sequence identity values are given in Table 2. DNA of *S*. *bovifelis*, *S*. *cruzi* and *S*. *hirsuta* extracted from individual sarcocysts were further used as positive or negative controls in the analysis of the digested samples. The control DNA sample of *S*. *hominis* was isolated from the muscular tissue of cattle in Italy [11] and subjected to* 18S* rRNA amplification and sequencing using the SarAF/SarBR and SarCF/SarDR primer pairs [23]. The obtained 1796-bp-long* 18S* rRNA sequence (MT792481) showed 99.4–99.7% similarity with *S*. *hominis* (JX679470–JX679471, KF954731) and up to 97.3% similarity with other *Sarcocystis* spp.

**Fig. 2:**
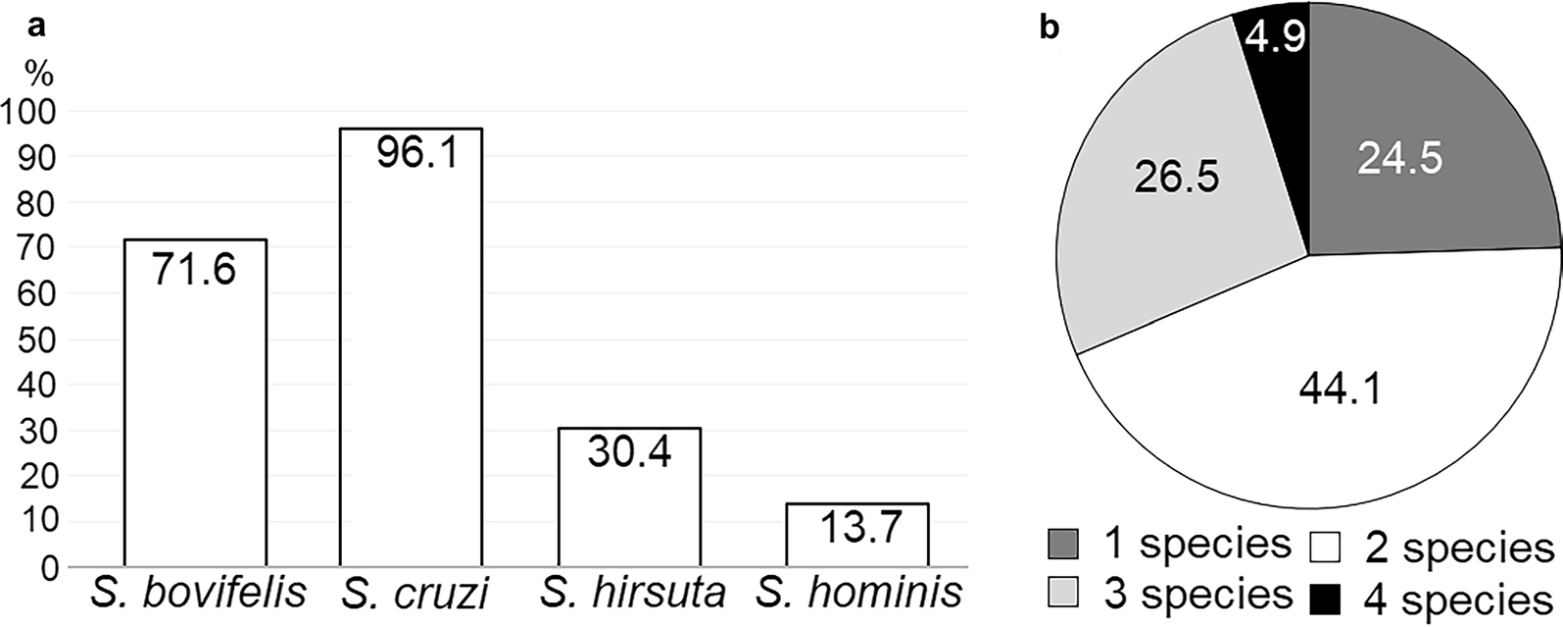
Identification of *Sarcocystis* spp. in digested muscle samples of cattle from Lithuania. **a** Prevalence of *Sarcocystis* species, **b** Distribution of the number of species in samples

*Sarcocystis* spp. were detected in all 102 beef diaphragm samples examined using the newly developed molecular identification technique based on muscle digestion and species-specific PCR analysis targeting *cox*1. The Lithuanian cattle analysed in this study were most frequently infected with *S*. *cruzi*, which was identified in 98 samples (96.1%) (Fig. 2a). The prevalence of *S*. *cruzi* in the analysed cattle was statistically significantly higher than that of *S*. *bovifelis* (71.6%; χ^2^ = 22.59, *P *< 0.0001), the presence of which was confirmed in 73 samples. The third most common species was *S*. *hirsuta* (30.4%), detected in 31 samples. The prevalence of *S*. *hirsuta* was statistically significantly lower than of *S*. *bovifelis* (χ^2^ = 34.60, *P *< 0.0001) and higher than that of *S*. *hominis* (13.7%; χ^2^ = 8.24, *P *< 0.01), which was detected in 14 samples. Although the molecular technique did not identify *S*. *hominis* among the individual sarcocysts excised from the diaphragm samples, it was identified in the digested samples. These results show that individual cattle may be infected up to four species of *Sarcocystis* at any one time (Fig. 2b). The most common finding was the presence of two species of *Sarcocystis* (45 samples, 44.1%); three species were detected in 27 samples (26.5%), a single species was identified in 25 samples (24.5%) and four species were identified in five samples (4.9%). In cases of a single species being detected, *S*. *cruzi* was identified in 21 samples and *S*. *bovifelis* was confirmed in four samples. When two species of *Sarcocystis* were observed in the samples, co-infection with *S*. *bovifelis*/*S*. *cruzi* was the most common finding (86.7%), whereas *S*. *cruzi*/*S*. *hirsuta* (8.9%) and *S*. *cruzi*/*S*. *hominis* (4.4%) co-infections were much rarer. Three different species were identified in 27 samples, with *S*. *bovifelis*/*S*. *cruzi*/*S*. *hirsuta* (74.1%) being the most common combination, while mixed infections with *S*. *bovifelis*/*S. cruzi*/*S*. *hominis* (18.5%) and *S*. *cruzi*/*S*. *hirsuta*/*S*. *hominis* (7.4%) were rarely detected. It is noteworthy that *S*. *hominis* was established in different districts of Lithuania: Pasvalys (two cases), Tauragė (two cases), Kupiškis (two cases), Anykščiai (two cases), Jonava (one case), Tauragė (one case), Šakiai (one case), Šilalė (one case), Skuodas (one case) and Kėdainiai (one case).

## Discussion

In the present study sarcocysts isolated from the diaphragm muscles of cattle were attributed to three morphological types, with molecular methods identifying the types as *S*. *cruzi*, *S*. *bovifelis* and *S*. *hirsuta*. Of the six *Sarcocystis* species known to use cattle as an intermediate host, two species, *S*. *cruzi* and *S*. *heydorni*, can be relatively easily differentiated by morphological characteristics [4]. Sarcocysts of *S*. *cruzi* are distinguished by hair-like protrusions, while sarcocysts belonging to *S*. *heydorni* have an apparently smooth cyst wall; sarcocysts of the other four species (*S*. *bovifelis*, *S*. *bovini*, *S*. *hirsuta* and *S*. *hominis*) are characterised by finger-like protrusions [4, 8, 9, 35]. During the course of this study, morphological examination of fragments of two sarocysts released from muscle fibres appeared to be smooth and to be similar to *S*. *heydorni*; however, based on molecular methods, these were identified as *S*. *cruzi*. In these cases, the protrusions of *S*. *cruzi* were likely torn off during manipulation of the sarcocysts with preparation needles. Moreover, sarococyts belonging to *S*. *bovifelis* were sometimes misidentified as *S*. *hirsuta* and* vice versa*. Sarcocysts of *S*. *hominis* were not found during the light microscopy study, but this species was confirmed in 13.7% of digested samples using molecular methods. These results suggest that the isolation of individual sarcocysts is ineffective when the prevalence of infection of the species is low and, therefore, that the digestion protocol may be preferable in epidemiological studies.

The* 18S* rRNA fragment used in the present study showed sufficient variability for the discrimination of *Sarcocystis* spp. found in cattle (Fig. 1a). Studies involving population genetic research on *Sarcocystis* species from cattle are scarce. One study demonstrated a low genetic variability among various isolates of *S*. *cruzi* from different geographical regions [36]. In the present study, analysis of *cox*1 sequences implied that the level of genetic variability depended on the *Sarcocystis* species and geographical area. Also, a relatively high genetic differentiation between the samples of compared species was revealed.

This study established that the GaBfEF/GaBfER, GaCrEF/GaCrER, GaHiEF/GaHiER and GaHoEF/GaHoER primer pairs were specific to *S*. *bovifelis*, *S*. *cruzi*, *S*. *hirsuta* and *S*. *hominis*, respectively. In all cases, no PCR product was generated using the negative DNA controls. In summary, the developed molecular technique based on the digestion of the muscle samples and species-specific PCR was found to be suitable for the detection of *S*. *bovifelis*, *S*. *cruzi*, *S*. *hirsuta* and *S*. *hominis* from beef. Methylene-blue staining of squash preparations of the muscle samples detected sarcocysts in 87.3% of preparations; in comparison, the molecular methods confirmed the presence of *Sarcocystis* spp. in all 102 of the digested specimens examined. A very high prevalence of *Sarcocystis* infection, exceeding 90%, has also been reported in cattle from Argentina, Belgium, the Czech Republic, Egypt, Germany, Iran, Italy, Japan, Mexico, New Zealand, Sudan, Thailand and Turkey (reviewed by Dubey et al. [1]). The findings of the present study are consistent with the data obtained by other researchers demonstrating a different infection prevalence depending on the method used. For example, Moré et al. [37] reported a higher level of prevalence when molecular methods were used (67.7% PCR, 69.6% real-time PCR) in comparison to microscopy analysis (40.0%). Daptardar et al. [38] calculated 44% infection prevalence using intact cyst isolation, 58% infection using pepsin-HCl digestion and 68% infection prevalence using PCR analysis. Hence, current *Sarcocystis* infection prevalence data should be considered as being critically dependent on the method employed to detect prevalence. The high prevalence of *Sarcocystis* spp. detected in cattle in Lithuania could be explained by husbandry practices, as carcasses of slaughtered cattle in small farms are fed to dogs and cats, especially in rural areas. Also, a raw diet, which has become popular among dog breeders in recent years, where pets are fed fresh meat, is one possible pathway for the spread of sarcocysts [39].

Our examination of the diaphragm samples of 102 cattle bred in Lithuania identified *S*. *cruzi* in 83.7% of samples, *S*. *bovifelis* in 71.6%, *S*. *hirsuta* in 30.4%, and *S*. *hominis* in 13.7% (Fig 2a). The prevalence and abundance of *Sarcocystis* species may depend on the type of the muscle being analysed. For example, *S*. *heydorni* tissue tropism was noted in cattle from China [35], with the highest prevalence (9.7%) recorded in skeletal muscles, followed by 3.4% prevalence in the oesophagus, 2.5% in the diaphragm and 0.1% in the tongue muscles; however, *S*. *heydorni* was absent in the heart muscle. This zoonotic species has also been identified in cattle from the Netherlands (1%) and Turkey [4, 10]. It should be noted that due to the different morphological, serological or molecular methods used in studies, it is difficult to compare the distribution of *Sarcocystis* species identified in cattle. Nevertheless, several trends in the prevalence of *Sarcocystis* species can be indicated. Most studies carried out in cattle reported the highest prevalence of *S*. *cruzi* employing canids as definitive hosts [10, 14, 15, 18, 37, 40–44]. By contrast, a relatively low prevalence of *S*. *hirsuta* using felids as definitive hosts was often recorded [14, 15, 37, 41, 45]. Research performed in cattle from the Netherlands and Italy showed *S*. *hirsuta* infection rates of < 2% [10, 40]. By contrast, using PCR-restriction fragment length polymorphism, *S*. *hirsuta* infection in Iran was recorded as reaching 58.9% [46], whereas microscopy analysis noted a prevalence of *S*. *hirsuta* as high as 94.0% in Brazil [47]. *Sarcocystis hirsuta* has been identified in different geographic regions [8, 10, 14, 15, 40, 44, 45]. Felids are also known to be definitive hosts of *S*. *bovifelis* and *S*. *bovini* [8, 48]; however, there is a lack of knowledge of the geographical distribution of these two *Sarcocystis* species. It should be pointed out that *S*. *bovini* has not been identified in Europe yet; *S*. *bovifelis* and *S*. *bovini* have been confirmed in Argentina, and only *S*. *bovini* has been found in New Zealand [8] and in beef from New Zealand imported to Japan [15]. In addition, based on comparison of* 18S* rRNA sequences *S*. *bovini* has also been found to be common in China [8]. Differences in the distribution of *Sarcocystis* species in cattle across geographical regions are possibly related to the abundance of definitive hosts.

We detected from one single *Sarcocystis* species up to four *Sarcocystis* species simultaneously in individual samples, with the highest prevalence noted for two species concurrently in one animal (Fig 2b). Two species forming sarcocysts in cattle, namely *S*. *bovifelis* and *S*. *bovini*, were (re)described only in 2016 [8]. Therefore, there is a lack of reliable data on the number of *Sarcocystis* species in individual bovine samples. However, mixed infections of different *Sarcocystis* species in cattle is a common occurrence [10, 37, 44]. Further studies are needed to reveal the rate of combinations of *Sarcocystis* species in individual samples from different countries.

The high level (> 40%) of *S*. *hominis* infection reported in cattle from countries such as Italy and Iran [33, 46] is most likely overestimated due to the confusion in identifying *S*. *hirsuta* and *S*. *bovifelis*/*S*. *bovini*. The majority of the* 18S* rRNA sequences formerly presented in GenBank as *S*. *hominis* actually belonged to *S*. *bovini*, *S*. *bovifelis* or *S*. *sinensis* infecting the water buffalo [8]. There is a lack of detailed *S*. *hominis* molecular data [49] and the first *cox*1 sequence of this species became available in the GenBank database only in 2018. The prevalence of *S*. *hominis* infection in different countries based on molecular methods is poorly studied. A 0.2% prevalence of *S*. *hominis* was detected in China [50], 0.5% in Argentina [42], 3.5% in Iran [44], 6.2 % in Germany [37] and 12.5% in the Netherlands [10]. The molecular technique established in this study is an important step in the development of zoonotic *S*. *hominis* diagnosis in both cattle and humans.

## Conclusion

Based on molecular methods, the presence of *S*. *cruzi* (96.1%), *S*. *bovifelis* (71.6%), *S*. *hirsuta* (30.4%), and *S*. *hominis* (13.7%) was confirmed in Lithuanian cattle meat for the first time. A rapid, accurate and relatively cheap technique was developed for the identification of *Sarcocystis* species in cattle meat. Further research on *S*. *hominis* epidemiology in various regions is needed.

## Data Availability

The sequences generated in the present study are available in GenBank database with accession numbers MT792432–MT792481 (*18S* rRNA) and MT796903–MT796964 (*cox*1).
